# Optimizing nanosilver for implant success: from marketing hype to medical reality

**DOI:** 10.1007/s11051-024-06181-2

**Published:** 2024-11-19

**Authors:** Georgios A. Sotiriou

**Affiliations:** 1 Department of Microbiology, Tumor and Cell Biology, Karolinska Institutet, 171 17 Stockholm, Sweden; 2 Department of Materials and Environmental Chemistry, Stockholm University, 106 91 Stockholm, Sweden

**Keywords:** Silver nanoparticles, Medical devices, Antibacterial, Antimicrobial resistance

## Abstract

Bacterial infections leading to implant failure pose a significant global health issue. Despite its antimicrobial properties, nanosilver is not commonly used in commercially available titanium implant coatings. This underutilization stems from an insufficient understanding of fundamental factors, such as particle size, coating, composition, and stability that dictate the antimicrobial performance of nanosilver coatings. A deeper understanding of these factors is crucial for designing effective nanosilver coatings to prevent biofilm formation on implants. Without this knowledge, nanosilver technology risks being merely a marketing tool rather than a functional component in medical devices. Another limiting factor is the potential cytotoxicity of nanosilver coatings, which necessitates a delicate balance between anti-biofilm activity and host tissue toxicity. Addressing these issues could involve the development of multifunctional coatings as well as the optimization of manufacturing processes with a specific focus on the durability of the coatings. Furthermore, to demonstrate the efficacy of these coatings, rigorous in vitro and in vivo assessments are required. As our understanding of the fundamental parameters of nanosilver coatings improves and we find ways to mitigate their toxicity, their utilization will be strengthened by clinicians and approved by regulatory agencies. The development of personalized implant coatings with well-defined nanosilver properties and multiple functionalities will further advance the field and address the challenge of implant failure.

## Main text

Implant failure from bacterial infections constitutes a major medical challenge as it often requires revision surgeries to several thousand patients per year globally. This results in immense costs for the healthcare system in addition to the psychological burden for the patients. State-of-the-art implants typically are made from titanium (Ti) and its alloys, because this material provides the necessary mechanical stability as well as a biocompatible surface for osseointegration. However, even when all precautions are taken by the clinical personnel, sometimes exogenous bacteria enter the surgical wound and might colonize the implant surface. Such bacteria may grow in colonies that are linked together with an extracellular matrix consisting of polysaccharides, proteins, DNA, and other biomolecules, forming a biofilm [1]. Biofilm infections are very difficult to treat, primarily because of the presence of this dense extracellular matrix that does not allow for any administered antibiotic drug to diffuse through and reach the bacteria. Such biofilms may be formed on both orthopedic implants (hip replacements, screws), as well as dental implants in the oral cavity, even though from different bacterial species in each category [2]. When the biofilm consists of bacteria that are resistant to current antibiotics and reach the bloodstream, there is a major risk for bacteremia and sepsis that may eventually lead to the death of the patient, especially if they belong to an immunocompromised patient group (e.g., diabetic, cancer patients). With the aging population globally increasing, it is predicted that the number of patients requiring implant placement will only increase in the next years. Therefore, there is a clinical and societal need to inhibit bacterial biofilm growth on the surface of implants that has yet to be met in clinical practice.

The low antibiotic drug diffusion through biofilms has sparked a significant amount of research in the last two decades to find antibiotic-free strategies to tackle this challenge [1]. Furthermore, the highest risk of infection lies during the first few days of the surgical wound from exogenous bacteria during the inflammatory phase of wound healing. Thus, there is a strong focus on developing anti-biofilm strategies to prevent bacterial growth during the first days after the implant placement. One way to do so is to develop coatings on the implant surface that exhibit intrinsic antimicrobial properties [1]. Such coatings are often fabricated in the nanoscale to increase their surface-to-volume ratio and enhance their antimicrobial activity. One of the most well-known nanomaterials with intrinsic antimicrobial properties is nanosilver (silver nanoparticles). Nanosilver exhibits a broad antimicrobial activity against bacteria, fungi, and viruses [3–5]. The main way this occurs is through the Ag^+^ ion release from the nanoparticles in aqueous environments, even though direct contact between the pathogen and the nanoparticle surface also plays a role [6, 7]. There is an intrinsic oxide layer (Ag_2_O) on the nanosilver that is the source of the released Ag^+^ ions through oxidative dissolution upon its contact with aqueous media [8]. This oxide layer dissolves rather quickly (hours to days) providing a high local Ag^+^ ion concentration during this period of time [9]. The antimicrobial mechanism of Ag^+^ ions against both planktonic bacteria and biofilms largely occurs through the generation of reactive oxygen species (ROS) that can bind to the bacterial membrane and its envelope, as well as disrupt protein and DNA functions [10], even though this activity is not selective to these compounds. This broad antimicrobial activity of nanosilver renders it a powerful material in biomedicine.

Several strategies exist for the fabrication of nanosilver coatings on surfaces, and upon performing a literature search in databases, a plethora of published articles appear that develop silver nanoparticles, deposit them on surfaces, and study their anti-biofilm effect [2, 11]. So, one would wonder: “if nanosilver is such a powerful biomaterial and can inhibit the growth of bacterial biofilms on implants, why is this material not routinely used as a coating on Ti implants to eradicate any bacteria present at the surgical wound and prevent implant failure by infections?” The answer to this paradox is multi-faceted. First, even though in the last years some commercially available Ti implants with nanosilver coatings have emerged, clinicians are not fully convinced yet of their effectiveness against biofilms. In fact, the lack of evidence in clinical practice for the undeniable superiority of nanosilver-coated implants suggests that there is still a lack of understanding of the important parameters for the success of such a product. Nanosilver is a very powerful antimicrobial material, but the antimicrobial performance of nanosilver depends on several parameters, such as its size, its coating, and its colloidal stability, to name a few. For example, a 5-nm nanosilver particle will exhibit drastically different bioactivity than a 20-nm particle [12], while a surface coating that is often applied on nanosilver particles may affect the Ag^+^ ion release profile to a large extent [13, 14]. Therefore, it is not enough to merely add nanosilver as a component in surface coatings of Ti implants, but it is crucial also to tailor the physicochemical properties of nanosilver to maximize its antimicrobial effect. Unless we obtain first a fundamental understanding of how and at what conditions such nanosilver coatings exhibit superior anti-biofilm activity and then utilize this understanding to design highly effective nanosilver coatings, this technology will mostly serve as a marketing tool instead of a functional component in medical devices.

Another reason for the limited employment in clinical practice of Ti implants with nanosilver coatings is the potential toxicity aspects. Both the released Ag^+^ ions and also the particles themselves may exhibit cytotoxicity to mammalian cells [12]. This may result in poor osseointegration with the host tissue and eventually implant failure. Therefore, it is extremely important to find an appropriate balance between the desirable anti-biofilm activity and any potential toxicity to the host tissue. One way to do so is to design coatings that exhibit strong antibacterial activity during the inflammation phase of wound healing but minimal activity during the proliferative phase [15]. Another way is to design multi-functional coatings and combine nanosilver with an osteogenic material, such as calcium phosphate or bioglass nanoparticles that exhibit both antibacterial activity as well as promote osseointegration [16]. Ultimately, an ideal nanosilver-based implant coating would exhibit the desired antimicrobial activity only in the presence of bacteria, on-demand in a self-triggered way. To do so, novel compositions relying on the fundamental understanding of both the Ag^+^ ion release physicochemical mechanism as well as the bacterial biofilm microenvironment are needed. Such a strategy has recently been demonstrated with alloyed Ag-Au nanostructured coatings, an otherwise rather inert material [17] that only releases the antimicrobial Ag^+^ ions in acidic conditions that are often present in the biofilm microenvironment [18]. The addition of Au during the synthesis of nanosilver results in Ag-Au nanoalloys, in which the presence of Au reduces the oxidation state of Ag [17–19], resulting in decreased Ag^+^ ion release through oxidative dissolution in neutral pH values. However, in low pH values, the less noble metal dissolves releasing Ag^+^ ions. This “dealloying” is a well-known process for the fabrication of nanoporous gold structures, often used in catalysis [20].

Another challenge in this field is the limited options for the reproducible and scalable nanomanufacturing of such coatings at a reasonable cost. Especially if coatings with multiple components and functionalities are required, as discussed above, there are not many options for their manufacturing that can yield high-quality and highly homogeneous coatings with reproducible characteristics among different batches. This is one of the major bottlenecks today in the nanomedicine field, with only a handful of nanomanufacturing processes that can deliver these advantages. One of these processes is flame aerosol technology that can yield high-quality, homogeneous coatings on surfaces in a rapid, reproducible, and inexpensive way [21]. Additive manufacturing is also an emerging process that may be able to address the above challenges in a personalized manner for each patient [22]. The mechanical stability of the coatings is also an important parameter that needs to be addressed. The coatings have to be highly durable with high cohesion and adhesion to the substrate in order to withstand the high mechanical forces that the implant experiences during its placement and during its osseointegration [23]. This requires additional considerations during the design of the implant, often overlooked in the scientific literature.

Finally, before implants with nanosilver coatings become the first choice for clinicians, rigorous in vitro and in vivo assessments need to be performed. Often, in vitro biofilm testing against a single bacterial species is enough for the first proof of concept as well as optimization of the physicochemical parameters of the developed coatings. However, the anti-biofilm efficacy needs to be demonstrated not only with site-specific bacteria (different bacteria cause biofilm infections in dental implants than orthopedic implants, for example), but also with multi-species bacteria [24] that are often more clinically relevant. Upon successful results in vitro, additional in vivo experiments for both the anti-biofilm performance as well as the osseointegration would be required. However, in vivo experiments to assess the antimicrobial effect of nanocoatings on titanium alloys are not trivial. Ideally, the antimicrobial activity of nanosilver coatings on titanium alloys would be examined with animal models in which nanosilver-modified titanium alloy structures would be inserted in the femoral medullary cavity or in tooth extraction sites, subsequently infected with the relevant pathogens and then monitored for specific durations to evaluate the final outcome [25]. These procedures are complicated and require expert personnel, rendering them rare and expensive. Nonetheless, such experiments would demonstrate further the effectiveness of the coatings, as the host immune system response also plays a role in fighting bacterial infections, and this parameter is absent when testing the efficacy in vitro.

Nanosilver coatings on medical implants have already emerged in the market. However, improvements in understanding the fundamental parameters that dictate the bioactivity of nanosilver coatings while simultaneously mitigating any undesired toxicity will further strengthen their utilization by clinicians and convince regulatory agencies of their safe and effective employment. This will be aided by the development of highly effective, reproducible, and inexpensive personalized implant coatings with well-defined nanosilver properties. Finally, nanosilver coatings that exhibit multiple functionalities and not only prevent biofilm infections but also promote osseointegration will advance the field beyond the state of the art and tackle the unmet societal and clinical challenge of implant failure.

## Data Availability

No datasets were generated or analysed during the current study.
